# The Effects of Developmental Stress on Dopaminergic Function in Adulthood: A Systematic Review and Meta-Analysis

**DOI:** 10.1192/bjo.2024.219

**Published:** 2024-08-01

**Authors:** Alexander Noar, George Chapman, Tinya Chang, James Payne-Gill, Michael Bloomfield

**Affiliations:** 1 University College London, London, United Kingdom; 2 Camden and Islington NHS Foundation Trust, London, United Kingdom

## Abstract

**Aims:**

Exposure to traumatic experiences during childhood and adolescence is a significant risk factor for the development of psychiatric disorders in adulthood. An estimated 50% of the worldwide incidence of depression and anxiety can be attributed to childhood maltreatment (Li et al., 2016). In addition, approximately one-third of psychotic experiences are attributable to a history of developmental trauma (McGrath et al., 2017). It is thought that long-lasting, trauma-induced adaptive changes in neurobiological function may lead to a predisposition towards pathophysiology (McCrory and Viding, 2015). However, the precise mechanisms through which developmental trauma exposure alters brain function on cellular and circuit levels remain poorly elucidated.

**Methods:**

A systematic literature search and meta-analysis was performed to establish how dopaminergic functioning in adulthood is affected by developmental stress in rodents. Three databases, Medline®, Embase®, and PsycINFO®, were systematically searched initially on 2nd December 2023. Terms for three superordinate concepts (‘childhood’ terms, ‘trauma’ terms, and ‘dopamine’ terms) were combined. Cohen's d statistic was used for effect sizes. This protocol is pre-registered on PROSPERO® (ID: CRD42018106382).

**Results:**

A total of 104 studies met our inclusion criteria. Meta-analysis indicated that developmental stress exposure leads to complex and long-lasting effects in basal and post-amphetamine extracellular dopamine concentrations in the medial prefrontal cortex, amygdala, and nucleus accumbens. In addition, there is a significant downregulation of D1 receptors and upregulation of D2 receptors in prefrontal and striatal regions involved in threat and reward processing. Effect sizes ranged from 0.36 to 1.55.

**Conclusion:**

These findings strongly suggest that dopaminergic dysfunction is a mechanistic link between developmental trauma and vulnerability towards mental illness in adulthood.

